# Sprouty genes are essential for the normal development of epibranchial ganglia in the mouse embryo

**DOI:** 10.1016/j.ydbio.2011.07.024

**Published:** 2011-10-01

**Authors:** Subreena Simrick, Heiko Lickert, M. Albert Basson

**Affiliations:** a Department of Craniofacial Development, King's College London, Floor 27, Guy's Tower, London, SE1 9RT, United Kingdom; b Helmholtz Zentrum München, Institute of Stem Cell Research, Ingolstädter Landstrasse 1, 85746 Neuherberg, Germany

**Keywords:** Sprouty, Cranial nerves, Epibranchial placodes, FGF8

## Abstract

Fibroblast growth factor (FGF) signalling has important roles in the development of the embryonic pharyngeal (branchial) arches, but its effects on innervation of the arches and associated structures have not been studied extensively. We investigated the consequences of deleting two receptor tyrosine kinase (RTK) antagonists of the Sprouty (Spry) gene family on the early development of the branchial nerves. The morphology of the facial, glossopharyngeal and vagus nerves are abnormal in *Spry1−/−;Spry2−/−* embryos. We identify specific defects in the epibranchial placodes and neural crest, which contribute sensory neurons and glia to these nerves. A dissection of the tissue-specific roles of these genes in branchial nerve development shows that Sprouty gene deletion in the pharyngeal epithelia can affect both placode formation and neural crest fate. However, epithelial-specific gene deletion only results in defects in the facial nerve and not the glossopharyngeal and vagus nerves, suggesting that the facial nerve is most sensitive to perturbations in RTK signalling. Reducing the *Fgf8* gene dosage only partially rescued defects in the glossopharyngeal nerve and was not sufficient to rescue facial nerve defects, suggesting that FGF8 is functionally redundant with other RTK ligands during facial nerve development.

## Highlights

► Sprouty gene deletion in mouse embryos cause multiple defects in epibranchial ganglia. ► Both epibranchial placodes and neural crest cells are affected. ► Sprouty gene function is required in the pharyngeal ectoderm and endoderm. ► Hyperactive FGF8 signalling is in part responsible for these defects.

## Introduction

The sensory innervation of the pharynx is provided by the epibranchial ganglia: the geniculate, petrosal and nodose. These neurons convey viscerosensory and gustatory information from the pharynx to the brainstem. The geniculate is the distal ganglion of the VIIth cranial nerve, the petrosal the distal ganglion of the IXth and the nodose the distal Xth. Importantly, the neurons are not neural crest derived but arise from discrete thickenings of the cranial ectoderm, the neurogenic placodes. The placodes that form these neurons are the epibranchial placodes and these structures develop in close apposition to the pharyngeal arches. The geniculate forms at the anterodorsal margin of the second arch, the petrosal at the third arch and the nodose placodes form alongside arches 4 and 6. Somatosensory innervation for the pharynx arises from the neural crest-derived proximal ganglia of these nerves (Baker and Bronner-Fraser, 2001; D'Amico-Martel and Noden, 1983; Ladher et al., 2010; Schlosser, 2010).

The formation of the epibranchial placodes is a multi-step process. Recent studies have demonstrated that the epibranchial and otic placodes are derived from a pre-otic field (or otic-epibranchial progenitor domain (OEPD)) and that this region is induced by FGF signals from the mesoderm and hindbrain. Several FGF ligands appear to function in a redundant fashion at this step of development in several species (Freter et al., 2008; Ladher et al., 2010; Sun et al., 2007; Urness et al., 2010). Experiments in zebrafish suggest that FGF signalling is required for the subsequent specification of the epibranchial placodes and for the development of the pharyngeal endoderm, which in turn provides appropriate signals that control neurogenesis in the placodes (Nechiporuk et al., 2005, 2007). Studies in the chick and zebrafish have suggested that secreted signals such as BMP7 from the endoderm regulate neurogenesis in the adjacent epibranchial placodes (Begbie et al., 1999; Kriebitz et al., 2009). Despite the abnormal segmentation of the pharyngeal apparatus observed in mouse embryos with reduced *Fgf8* expression (Frank et al., 2002), the branchial sensory ganglia are reported to be normal at E10.5 of development (Chi et al., 2003). In addition to the potential roles of FGF ligands in early branchial nerve development, other growth factors, such as Glial cell line-derived neurotrophic factor (GDNF) are expressed during epibranchial placode development (Homma et al., 2000; Kyuno and Jones, 2007; Suvanto et al., 1996).

Sprouty genes encode feedback antagonists of RTK signalling and embryonic defects in Sprouty-deficient embryos have been linked to increased FGF and RET signalling (Mason et al., 2006). A recent study has shown that the specification of the otic placode from the OEPD is limited by *Spry1* and *Spry2*: in *Spry1−/−;Spry2−/−* embryos, the otic placode is increased in size at the expense of the non-otic ectoderm, which presumably includes the precursors of the epibranchial placodes (Mahoney Rogers et al., 2011). However, the role of Sprouty genes in regulating the development of the epibranchial placodes has not been addressed. In order to investigate the effects of deregulated RTK signalling on the development of the branchial nerves in the mouse embryo, we simultaneously deleted two Sprouty genes during development. We provide evidence that Sprouty genes regulate RTK signalling during formation of the epibranchial placodes, neurogenesis within the placodes and neural crest fate specification. However, our data also suggests significant differences between the different epibranchial placodes with respect to the effects deregulated RTK signalling have on their development.

## Materials and methods

### Mouse lines and embryos

**Supplementary Fig. 1. Specificity of cre lines used in this study.**
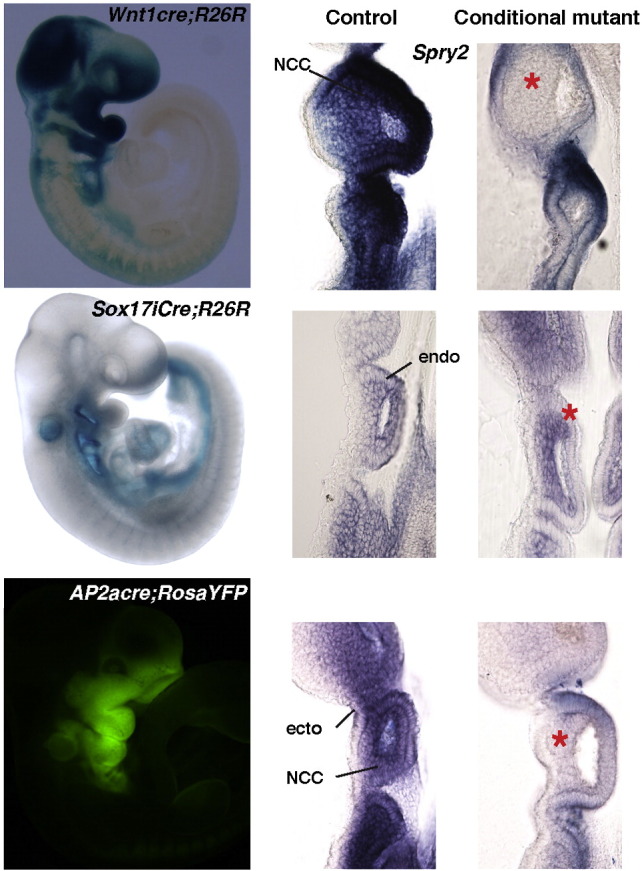
Cre reporter activity (R26R stained with X-gal (blue) or green fluorescence from RosaYFP reporter, as indicated) for the cre lines used in this study are shown in the left column. *Wnt1cre* and *Sox17iCre* embryos are E9.5 and the *AP2*α*cre* embryo is E10.5. *Spry2* expression as determined by in situ hybridisation on control (cre-negative) and conditional mutant E9.5 embryos, are shown in the two columns on the right. Note the loss of *Spry2* expression in neural crest cells (NCC) in the *Wnt1cre* conditional mutant, in the endoderm (endo) in *Sox17iCre* conditional mutants and in the ectoderm (ecto) + NCC in *AP2*α*cre* conditional mutants.

The mouse lines used in this study were maintained on a mixed genetic background and have been described previously: *β-actin-cre* (Lewandoski et al., 1997), *Ap2αcre* (Macatee et al., 2003), *Sox17i-2A-iCre* (*Sox17icre*) (Engert et al., 2009), *Wnt1cre* (Danielian et al., 1998), R26R (Soriano, 1999), *Spry1*^*tm1.1Jdli*^ (Basson et al., 2005) and *Spry2*^*tm1.1Mrt*^ (Shim et al., 2005). Mice carrying *β-actin-cre* were crossed with those carrying conditional *Spry1*^*flox*^ and *Spry2*^*flox*^ alleles to generate *Spry1*^*−*^and *Spry2*^*−*^null alleles. Embryos lacking both genes (*Spry1*^*−/−*^*;Spry2*^*−/−*^) were produced by crossing *βactinCre/βactinCre;Spry1*^*+/−*^*;Spry2*^*+/−*^ males with *Spry1*^*flox/flox*^*;Spry2*^*flox/flox*^ females. For rescue experiments, *βactinCre*^*2*^*;Spry1*^*+/−*^*;Spry2*^*+/−*^*;Fgf8*^*+/−*^ males were crossed with *Spry1*^*flox/flox*^*;Spry2*^*flox/flox*^ females using the *Fgf8*^*tm1.2Mrt*^ allele (Meyers et al., 1998). Tissue-specific mutants were produced by crossing *Cre;Spry1*^*flox/+*^*;Spry2*^*flox/+*^ males to *Spry1*^*flox/flox*^*;Spry2*^*flox/flox*^ females. Tissue specific cre recombinase activity was confirmed using the R26R (Soriano, 1999) or RosaYFP (Srinivas et al., 2001) reporter mouse lines and in situ hybridisation for *Spry1* or *Spry2* (Suppl. Fig. 1).

After timed matings, noon on the day of the vaginal plug was taken as E0.5. However, due to the rapid growth of the cranial nerves during development, embryos were staged more accurately using the number of somites. Harvested embryos were fixed overnight in 4% formaldehyde at 4 °C, dehydrated and stored in 100% MeOH at − 20 °C until whole mount or section anaylsis. For section analysis, embryos were embedded in paraffin and sectioned at 7 μm in preparation for immunohistochemistry.

### Genotyping

PCR amplification using DNA from embryonic yolk sac was used to genotype harvested embryos. With the exception of *Sox17icre*, all other cre lines were genotyped using the following PCR primers, CCT GGA AAA TGC TTC TGT CCG and CAG GGT GTT ATA AGC AAT CCC, which give a 390 bp product in cre positive embryos and no product in wild type embryos. *Sox17icre* genotyping was performed using primers GTG TAT AAG CCC GAG ATG G, CTC AAC TGT TCA AGT GGC AG and GAT CTA TGG TGC CAA GGA TGA C, to give a 446 bp cre product and a 288 bp wildtype product. *Spry1* genotyping primers are GGG AAA ACC GTG TTC TAA GGA GTA GC, GTT CTT TGT GGC AGA CAC TCT TCA TTC and CTC AAT AGG AGT GGA CTG TGA AAC TGC; PCR produces a 342 bp product with a flox allele, a 150 bp product with a null allele and a 311 bp product with a wild type allele. Genotyping primers for *Spry2* are GGA TGG CTC TGA TCT GAT CC, TTG AGA ACA TGC CTC GAC C and GCA TGG GCT ATT CAC AAA C, and resulting PCR products include a 500 bp product with a flox allele, a 225 bp product with a null allele and a 350 bp product with a wild type allele. *Fgf8* genotyping primers are CTA CCC ATC TTC CCC ACA AAA C and CCT GAA AAC TGA ACG CTG GTC C, where a PCR product of 1100 bp is obtained with a null allele and no product from a wild type allele.

### Whole mount and section in situ hybridisation

Whole mount RNA in situ hybridisation was performed according to standard protocols using Digoxigenin-labelled RNA probes. *Spry1*, *Spry2* (Minowada et al., 1999), *Sox10* (Calmont et al., 2009), *Crabp1* (Randall et al., 2009), *Ngn1* (Ma et al., 1996), *Ngn2* (Fode et al., 1998), and *Dlx2* (Depew et al., 2005) antisense RNA probes were produced from constructs as described in the original publications.

### Whole mount and section immunohistochemistry

Whole mount and section immunohistochemistry was performed using anti-neurofilament, RMO-270 (Zymed) at a dilution of 1/3000, with Alexa 488 goat anti mouse secondary antibody (Invitrogen) at a dilution of 1/200. Phox2b labelling of motor and sensory neurons was performed using rabbit anti-Phox2b (Pattyn et al., 1997) at a dilution of 1/800, with Alexa 546 goat anti rabbit secondary antibody (Invitrogen) at a dilution of 1/200 and counterstained with DAPI. Fluorescent pictures were rendered black and white using the gradient map tool in Adobe Photoshop to optimise visualisation in print.

In whole mount embryos individual epibranchial cranial nerves on each side of the embryo were considered separately when analysing phenotypic incidence (1 embryo = 2n) and significant difference probability values were calculated using two-tailed Fisher's exact test (Prism 5, Graphpad software Inc.).

### Pharyngeal arch measurements

**Supplementary Fig. 2:**
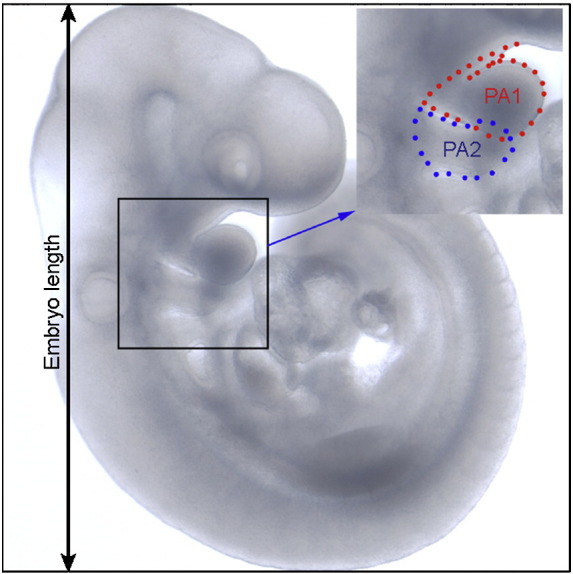
2D lateral pharyngeal arch measurements. E9.5 embryos were photographed using a Nikon SMZ1500 microscope at a resolution of 2560 × 1920 pixels. Images were cropped as indicated by the square around the embryo and the height of all embryo images set as 7 cm at 300dpi to allow a direct comparison to be made between embryos of slightly different stages of development and sizes. Adobe Photoshop (Adobe Systems Incorporated) was used to measure 2D pharyngeal arch area in pixels (as indicated in the inset: first pharyngeal arch in red, second pharyngeal arch in blue). Statistical analysis was performed using the D'Agostino and Pearson omnibus normality test, the unpaired two-tailed *t*-test and the two-tailed Mann–Whitney test as applicable (Prism 5, Graphpad software Inc).

2D lateral pharyngeal arch area was measured using pictures taken on a Nikon SMZ1500 microscope at a resolution of 2560 × 1920 pixels. Measurements were normalised to the total embryo length to account for variations in embryonic size as indicated in Suppl. Fig. 2. Adobe Photoshop (Adobe Systems Incorporated) was used to measure 2D pharyngeal arch area in pixels. Statistical analysis included the D'Agostino and Pearson omnibus normality test, the unpaired two-tailed *t*-test and the two-tailed Mann–Whitney test (Prism 5, Graphpad software Inc).

## Results

### Sprouty gene expression in relation to epibranchial placode development

**Fig. 1:**
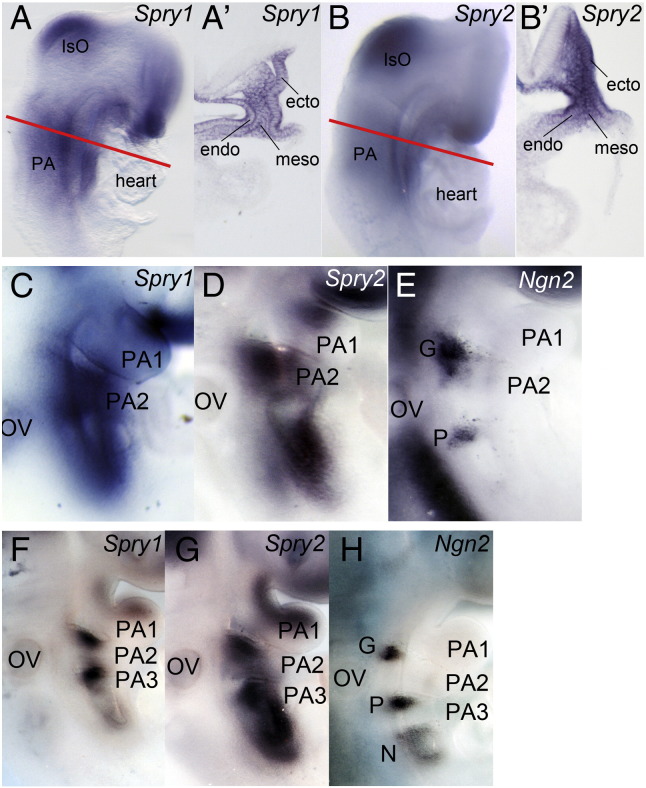
Dynamic *Spry1* and *Spry2* expression in the developing pharyngeal region. Sprouty gene expression analysed by RNA in situ hybridisation. *Spry1* and *Spry2* expression in whole mount E8.5 (8–9ss) (A,B), E9 (16–20ss) (C,D) and E9.5 (25–26ss) (F,G) embryos, anterior to the top and ventral to the right. Embryos in A and B were sectioned as indicated by the red line and sections are shown in A′ and B′, respectively. Note *Spry1* and *Spry2* expression in the ectoderm (ecto), mesoderm (meso) and endoderm (endo) at E8.5. *Ngn2* expression is shown to indicate the position of the epibranchial placodes at E9 (E) and E9.5 (H) for comparison. The first (PA1), second (PA2) and third (PA3) pharyngeal arches; otic vesicle (OV); geniculate (G), petrosal (P) and nodose (N) placodes are labelled.

To determine whether Sprouty genes may have a function in the development of the epibranchial ganglia in the mouse, we analysed the expression of *Spry1* and *Spry2* between embryonic day (E)8.5 and E9.5 of development. At E8.5, *Spry1* and *Spry2* are both expressed in the pharyngeal ectoderm, endoderm and mesoderm (Figs. 1A–B′). This pattern of expression is consistent with the expression of several Fgf genes in these tissues at this time of development and the role of FGF signalling in the induction of otic fate (Ladher et al., 2010).

Over the next day of development, *Spry1* and *Spry2* expression gradually becomes restricted to discrete regions in the pharyngeal arches. At E9, *Spry1* and *Spry2* are expressed in the regions where the *Ngn2*-positive geniculate and petrosal placodes are induced (Figs. 1C–E). At E9.5, Sprouty expression was observed ventral to the geniculate and broadly in a region that appears to overlap with the petrosal and nodose placodes (Figs. 1F–H). These data suggest that Sprouty genes may regulate RTK signalling during epibranchial placode specification and neurogenesis.

### Branchial nerve defects in Sprouty mutants

**Fig. 2:**
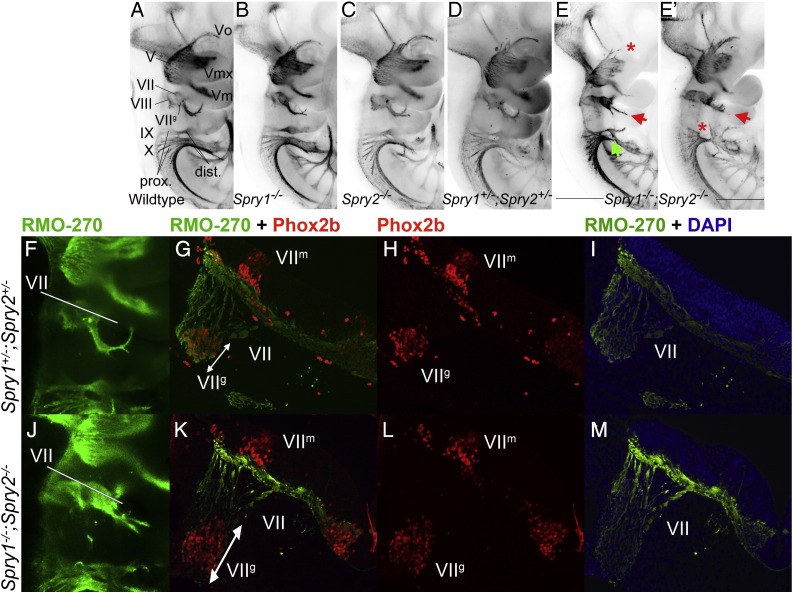
Cranial nerve abnormalities in Sprouty mutants. Anti-neurofilament immunohistochemistry revealing developing cranial nerves of wildtype (A), *Spry1*^*−/−*^ (B), *Spry2*^*−/−*^ (C), *Spry1*^*+/−*^*;Spry2*^*+/−*^ (D) and *Spry1*^*−/−*^*;Spry2*^*−/−*^ (E,E′) E10.5 embryos is shown. All embryos were between 34 and 36 somite stage. Standard labelling of the cranial nerves was used, trigeminal ganglion (V) with opthalmic (Vo), maxillary (Vmx) and mandibulary (Vm) branches, facial nerve (VII); vestibulocochlear nerve (VIII); glossopharyngeal nerve (IX) and the vagus nerve (X). Arrows highlight abnormal morphology and asterisks indicate missing portions. The majority of developing cranial nerves present in *Spry1*^*−/−*^ (B), *Spry2*^*−/−*^ (C) and *Spry1*^*+/−*^*;Spry2*^*+/−*^ (D) embryos were comparable to wildtype and the latter genotype was used as a control in this study. *Spry1*^*−/−*^*;Spry2*^*−/−*^ embryos have trigeminal defects (e.g. absent ophthalmic branch of the trigeminal nerve in E), facial nerve defects and glossopharyngeal and vagus cranial nerves display incomplete or irregular bridging between proximal and distal ganglia (E,E′). (F–M) Neurofilament (RMO-270) and Phox2b immunohistochemistry counter stained with DAPI on sections from E10.5 *Spry1*^*+/−*^*;Spry2*^*+/−*^ control (F–I) and *Spry1*^*−/−*^*;Spry2*^*−/−*^ (J–M) mutant embryos. Neurofilament staining is indicated with green fluorescence, Phox2b labelling of motor and sensory neuron nuclei in red and DAPI stained nuclei in blue. Labelling of markers and genotypes of merged images are as indicated. Other labels include, facial cranial nerve (VII) with motor nuclei in rhombomere 4 of the hindbrain (VII^m^) and sensory neuron nuclei in the developing geniculate ganglion (VII^g^). The white lines in F and J indicate plane of section for the images on the right. Motor nuclei are present in rhombomere 4 of the hindbrain in both the *Spry1*^*−/−*^*;Spry2*^*−/−*^ mutant and *Spry1*^*+/−*^*;Spry2*^*+/−*^ control embryos and are positioned adjacent to the developing geniculate ganglion (n = 4). The geniculate ganglion is enlarged in the *Spry1*^*−/−*^*;Spry2*^*−/−*^ embryos compared to the *Spry1*^*+/−*^*;Spry2*^*+/−*^ controls (compare white arrows in G with K).

Whole E10.5 embryos were stained with an antibody to neurofilament to reveal the developing cranial nerves (Figs. 2A–E′). Cranial nerves in *Spry1*^*−/−*^ (n = 9/10) and *Spry2*^*−/−*^ (n = 8/8) embryos exhibited normal morphologies when compared to wildtype controls (n = 10) at E10.5 (compare Figs. 2B and C with A, Table 1). Most *Spry*^*+/−*^*;Spry2*^*+/−*^ embryos (n = 28/34) also exhibited no cranial nerve defects, confirming that the loss of two Sprouty alleles is insufficient to cause significant cranial nerve defects (Fig. 2D). These observations suggest that *Spry1* and *Spry2* may functionally compensate for the loss of each other during branchial nerve development.

**Supplementary Fig. 3:**
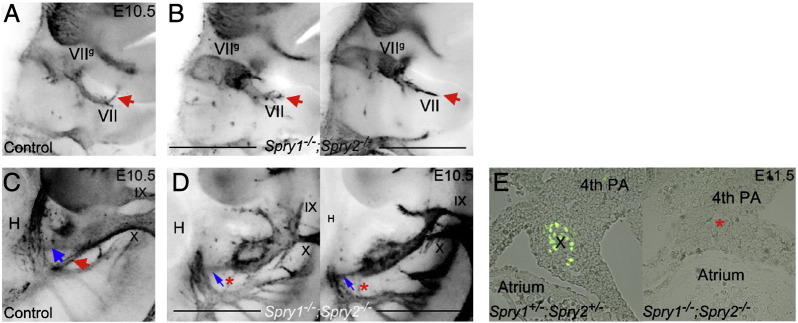
Analysis of *Spry1*^*−/−*^*;Spry2*^*−/−*^ facial and vagus nerve projections. The VIIth nerve penetrates the proximal second arch and turns anteriorly once it reaches the distal arch (red arrow) in control embryos (A). This nerve fails to turn in an anterior direction in *Spry1*^*−/−*^*;Spry2*^*−/−*^ embryos with variable branching patterns (red arrows in B). High magnification views of the ventral-posterior projections of the distal vagus nerve are shown in control (C) and mutant (D) embryos at E10.5. A ventral projection towards the heart (blue arrows) appears diminished in mutant embryos and a posterior projection towards the gut (red arrow) appears to be absent (red asterisks) in mutant embryos at E10.5. The vagus nerve projection to the heart can be visualised in sections through an E11.5 control embryo by neurofilament stain (green stain in E), which appears to be absent in sections through a mutant embryo (red asterisk). VII = facial nerve; VIIg = facial ganglion; IX = glossopharyngeal nerve and X = vagus nerve.

In agreement with this hypothesis, *Spry1*^*−/−*^*;Spry2*^*−/−*^ embryos (n = 36) displayed severe abnormalities in all the branchial nerves at E10.5 (Fig. 2E,E′, Table 1). An abnormally developing facial nerve (VII) was the most prevalent defect observed in *Spry1*^*−/−*^*;Spry2*^*−/−*^ embryos (n = 35/36). In control embryos, the VIIth nerve penetrated the proximal second arch and turned anteriorly once it reached the distal arch (Fig. 2A and Suppl. Fig. 3). Nerve projections from the facial ganglion into the second arch appeared stunted and failed to turn in an anterior direction (red arrow in Fig. 2E,E′, n = 34/36, two-tailed Fisher's exact text, p < 0.0001). In some cases, the VIIth nerve also exhibited severe defasciculation (Fig. 2E′, Suppl. Fig. 3).

To assess the size of the geniculate ganglion, we stained sections from E10.5 embryos (indicated by white lines in Figs. 2F,J) with antibodies to Phox2b and neurofilament (Figs. 2G–I, K–M). Phox2b is expressed in the facial motor neurons (VII^m^) in the hindbrain and epibranchial sensory neurons in the geniculate ganglion (VII^g^) (Pattyn et al., 1997). A normal distribution of Phox2b + motoneurons was present in rhombomeres 4 and 5 (r4/r5) of *Spry1*^*−/−*^*;Spry2*^*−/−*^ embryos compared to *Spry1*^*+/−*^*;Spry2*^*+/−*^ controls (Figs. 2G,H,K,L). By contrast, the geniculate ganglion was enlarged in *Spry1*^*−/−*^*;Spry2*^*−/−*^ embryos (Figs. 2G,H,K,L; n = 34/36, two-tailed Fisher's exact text, p < 0.0001). These observations suggest that the defects in the developing geniculate ganglion are not secondary to defects in hindbrain development.

The *Spry1*^*−/−*^*;Spry2*^*−/−*^ embryos also exhibited absent or diminished glossopharyngeal nerve fibres in 13/36 embryos (red asterisk, Fig. 1E′, two-tailed Fisher's exact text p = 0.0029). In the most severe cases, observed in 5/36 embryos, the early glossopharyngeal nerve appeared completely missing or fused with the vagus nerve. The vagus nerve (X) was also abnormal (9/36) or absent (11/36) in *Spry1*^*−/−*^*;Spry2*^*−/−*^ embryos (Fig. 1E, green arrow indicates abnormal region, n = 20/36, two-tailed Fisher's exact text p < 0.0001). In embryos where the vagus nerve could be distinguished, a closer examination of its projections suggested that projections towards the heart were diminished and that projections towards the gut were absent (Suppl. Fig. 3). Consistent with the observation that the vagus nerve appeared absent in most *Spry1*^*−/−*^*;Spry2*^*−/−*^ embryos at E10.5, no apparent vagus nerve projection into the heart was observed in a *Spry1*^*−/−*^*;Spry2*^*−/−*^ embryo examined at E11.5 (Suppl. Fig. 3).

In summary, defects could be detected in all branchial nerves of *Spry1*^*−/−*^*;Spry2*^*−/−*^ embryos, with defects in the glossopharyngeal and vagus nerves being less frequent.

### Defects in sensory placode formation in the absence of Sprouty genes

**Fig. 3:**
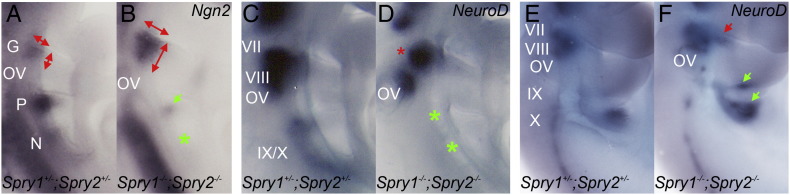
Defects in epibranchial placode formation and neuronal differentiation in Sprouty mutant embryos. Whole mount in situ hybridisation with a *Ngn2* RNA probe (A,B) to detect the developing placodes and a *NeuroD* RNA probe (C–F) to identify differentiating neuroblasts. Arrows indicate changes in gene expression and asterisks highlight regions where gene expression had been lost. Annotations are the same as in Figs. 1 and 2; with the vestibulo-acoustic nerve (VIII) also indicated. Note the enlarged geniculate placode in the *Spry1*^*−/−*^*;Spry2*^*−/−*^ E9.5 embryos compared to *Spry1*^*+/−*^*;Spry2*^*+/−*^ controls (A,B) (n = 6). Conversely, note the smaller or absent petrosal and nodose placodes in the *Spry1*^*−/−*^*;Spry2*^*−/−*^ embryos. *NeuroD* expression is reduced at E9.5 (C,D) (n = 4). *NeuroD* expression recovers and appears increased in the geniculate (red arrow), geniculate and petrosal (green arrows) ganglia of *Spry1*^*−/−*^*;Spry2*^*−/−*^ embryos compared to *Spry1*^*+/−*^*;Spry2*^*+/−*^ controls (n = 4) at E10.5 (E,F).

One of the first markers for epibranchial placode specification is the proneural gene, *Ngn2* (Fode et al., 1998; Sommer et al., 1996). To assess whether the specification of individual epibranchial placodes was compromised in the Sprouty mutants, whole mount in situ hybridisation using a *Ngn2* riboprobe was performed at E9.5 (Fig. 3A). *Ngn2* expression was expanded at the anterodorsal margin of the second arch, indicating an enlargement of the geniculate placode in the *Spry1*^*−/−*^*;Spry2*^*−/−*^ embryos (Fig. 3B; n = 4). This is in line with the observation of a larger facial ganglion in *Spry1*^*−/−*^*;Spry2*^*−/−*^ embryos at E10.5 (Fig. 2). By contrast, the petrosal and nodose placodes appeared smaller or missing in the *Spry1*^*−/−*^*;Spry2*^*−/−*^ mutants at E9.5 (Fig. 3B; n = 4). These observations suggest that increased RTK signalling has opposite effects on the geniculate versus petrosal and nodose placodes at the time of their formation.

After the specification of the epibranchial placodes and initiation of *Ngn2* expression, neuronal precursors delaminate from the placodes and start expressing *NeuroD* as they migrate towards the developing ganglia (Fode et al., 1998). Despite the enlarged geniculate placode, the expression of *NeuroD* was reduced in the geniculate ganglion of *Spry1*^*−/−*^*;Spry2*^*−/−*^ embryos compared to the *Spry*^*+/−*^*;Spry2*^*+/−*^ controls (red asterisk, Fig. 3D; n = 4). This defect appears to be temporary, as *NeuroD* expression appears slightly increased or expanded by E10.5 (red arrows, Fig. 3F; n = 4). A more pronounced effect was observed in the developing glossopharyngeal (IX) and vagus (X) ganglia, where *NeuroD* expression appeared missing at E9.5 (green asterisks, Fig. 3D) (n = 4), but were present at increased levels by E10.5 (green arrows, Fig. 3E; n = 4).

Taken together, these data indicate that initial placode formation is affected by the absence of *Spry1* and *Spry2*, with the geniculate placode increased in size and the petrosal and nodose reduced. Neuronal differentiation as measured by *NeuroD* expression is reduced in all epibranchial ganglia at E9.5, but this effect appears to be transient with *NeuroD* expression increased by E10.5. These observations suggest a transient delay in neuronal differentiation in Sprouty mutant embryos.

### The second pharyngeal arch is enlarged in Sprouty-deficient embryos

During the course of our investigation, we noticed that the increase in the geniculate placode appeared to correlate with an enlargement of the second pharyngeal arch itself. Sizes of the first and second pharyngeal arches were measured in E9.5 embryos using lateral 2D surface area, and total embryo length was used to standardise measurements (Table 2). Statistical analysis was performed using Prism 5 (Graphpad software Inc.) and significant differences were calculated using parametric and non-parametric tests depending on whether the measurement sets passed the D'Agostino and Pearson test for normality. No significant difference was found between the first pharyngeal arch of *Spry1*^*−/−*^*;Spry2*^*−/−*^ (n = 36) embryos and *Spry*^*+/−*^*;Spry2*^*+/−*^ (n = 36) controls (unpaired two-tailed *t*-test, p = 0.1929). However, a significant difference was found between the second pharyngeal arch sizes of the mutant and control embryos (n = 36 for both genotypes) (unpaired two-tailed *t*-test, p < 0.0001). This data confirms that the second pharyngeal arch was significantly larger in *Spry1*^*−/−*^*;Spry2*^*−/−*^ embryos, whilst the first arch was of normal size. These observations suggest that the enlargement of the geniculate may be secondary to a more general pharyngeal defect that results in abnormal growth of the second pharyngeal arch.

### Altered neural crest fate in Sprouty-deficient embryos

It has been shown that neural crest cells are not required for the formation of the epibranchial placodes, but that they play an important role in directing the migration of epibranchial placodal cells inwards to the site of ganglion formation (Begbie and Graham, 2001; Coppola et al., 2010). Furthermore, recent experiments in chick embryos have shown that the inhibition of FGF signalling is also associated with the failure of neural crest cells to turn on markers of ectomesenchymal fate when entering the pharyngeal arches; instead, these cells retained markers of nonectomesenchymal neural crest (*Sox10*, *Foxd3*) which includes cells that contribute to the sensory ganglia (Blentic et al., 2008). We wished therefore to determine if deletion of Sprouty genes had consequences for the development of the neural crest cells and more specifically whether neural crest cell fate was altered upon the loss of Sprouty genes. We hypothesised that the loss of FGF antagonists would result in the premature expression of ectomesenchymal fate markers at the expense of non-ectomesenchymal markers. We therefore used a number of neural crest markers to visualise these cells in the developing embryo.

**Fig. 4:**
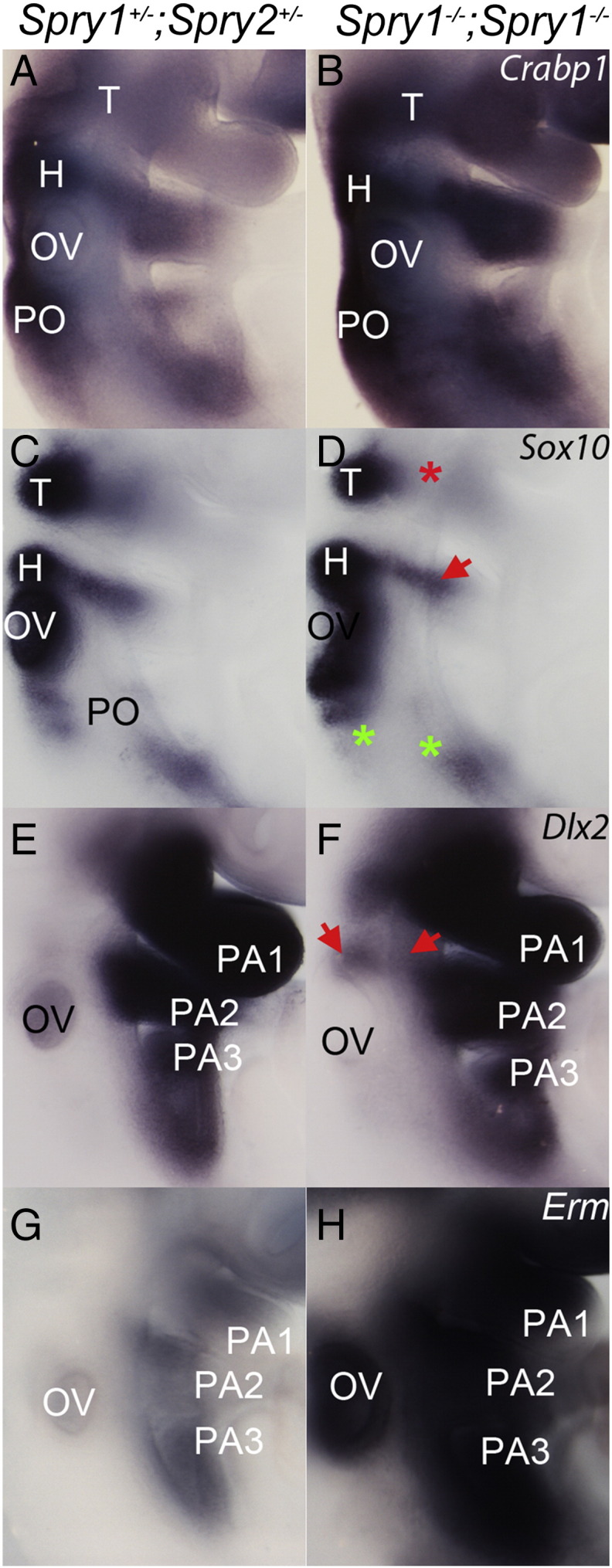
Neural crest cell fate appears altered in *Spry1*^*−/−*^*;Spry2*^*−/−*^ embryos. Whole mount in situ hybridisation of control (A,C,E,G) and *Spry1*^*−/−*^*;Spry2*^*−/−*^ embryos (B,D,F,H) with neural crest markers *Crabp1* (A,B) (n = 6), *Sox10* (C,D) (n = 4), *Dlx2* (E,F) (n = 4), and a reporter of FGF signalling *Erm* (G,H) all at E9 (20–22 somite stage). Neural crest streams, pharyngeal arches and otic vesicle are labelled as in Figs. 1 and 2. Arrows illustrate changes in gene expression and asterisks indicate regions with absent gene expression.

Whole-mount in situ hybridisation for a pan-neural crest marker *Crabp1* showed that all the neural crest streams were present and migrating to the appropriate regions in the *Spry1*^*−/−*^*;Spry2*^*−/−*^ mutants (Figs. 4A,B; n = 8). To distinguish between ectomesenchymal and non-ectomesechymal neural crest cells we used *Dlx2* and *Sox10* as respective markers (Baker et al., 1997; Blentic et al., 2008). The intensity of *Sox10* expression where the hyoid neural crest stream approaches the geniculate placode was slightly reduced in *Spry1*^*−/−*^*;Spry2*^*−/−*^ mutants (red arrow, Figs. 4C,D; n = 8). Intriguingly, *Dlx2* expression appeared to respond in the opposite manner and was markedly upregulated in this region (red arrows, Figs. 4E,F; n = 4). These gene expression changes are consistent with a change in neural crest cell fate from non-ectomesenchymal to ectomesenchymal in the vicinity of the geniculate placode. *Sox10* expression was also decreased in the post otic stream where the petrosal and nodose placodes develop (green asterisks, Figs. 4C,D; n = 8), although no corresponding up-regulation of *Dlx2* expression in the third and developing fourth pharyngeal arches was observed (Figs. 4E,F; n = 4).

To confirm that this apparent switch in neural crest cell fate is associated with increased RTK signalling, we determined the expression of *Erm (Etv5),* a downstream target of FGF signalling (Klein et al., 2008). *Erm* was upregulated in the *Spry1*^*−/−*^*;Spry2*^*−/−*^ mutants compared to controls, including in regions where the altered *Sox10* and *Dlx2* expression was detected (Figs. 4G,H; n = 8). These findings are consistent with previous experiments in the chick embryo showing that increased FGF signalling promoted ectomesenchymal fate at the expense of neurogenic fate (Blentic et al., 2008).

### Epithelial Sprouty gene expression regulates neural crest contribution to the forming facial ganglia

**Fig. 5:**
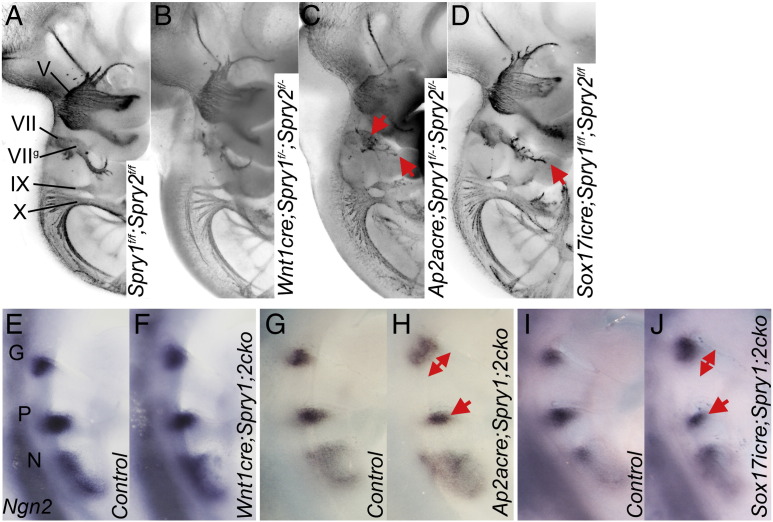
Sprouty function in the pharyngeal epithelia is required for normal facial nerve morphogenesis. (A–D) Anti-neurofilament (RMO-270) whole mount immunohistochemistry revealing cranial nerve morphology in *Spry1* and *Spry2* tissue specific conditional knockout E10.5 embryos. Tissue specific gene deletion was achieved using the cre-lox system with *Spry1* and *Spry2 flox* alleles and *Wnt1cre* (B, neural crest), *Ap2αcre* (C, ectoderm and neural crest), and *Sox17icre* (D, endoderm). A stage-matched *Spry1*^*f/+*^*;Spry2*^*f/+*^ litter-mate control is displayed to indicate normal cranial nerve morphology (A). Genotypes are as indicated and labels are as before. Red arrows illustrate changes in cranial nerve morphology. (E–J) Whole mount in situ hybridisation with *Ngn2* antisense RNA probe to reveal the epibranchial placodes in *Wnt1cre-, AP2acre-,* or *Sox17icre-*mediated conditional knockout (cko) embryos as indicated. Note the enlarged geniculate placode and slightly smaller petrosal in the *Ap2αcre;Spry1*^*f/−*^*;Spry2*^*f/−*^and *Sox17cre;Spry1*^*f/f*^*;Spry2*^*f/f*^ embryos (red arrows in H and J). The epibranchial placodes, geniculate (G), petrosal (P) and nodose (N) are labelled.

We next asked whether the defects in neural crest and placodal development were cell autonomous in *Spry1*^*−/−*^*;Spry2*^*−/−*^ mutants. *Spry1* and *Spry2* were excised in different tissues using several cre lines. No defects were detected in E10.5 *Wnt1cre;Spry1*^*f/−*^*;Spry2*^*f/−*^embryos (n = 8), indicating that the loss of Sprouty genes in the neural crest is not sufficient to cause cranial nerve defects (Figs. 5A,B, Table 1). Furthermore *Ngn2* expression was normal in the epibranchial placodes of these mutants (Figs. 5E,F; n = 4), indicating that defects in the development of the epibranchial placodes were not caused by defects in the neural crest alone.

To determine whether the loss of *Spry1* and *Spry2* in the pharyngeal ectoderm is sufficient to cause defects in placodal neurogenesis, we deleted Sprouty genes from the pharyngeal ectoderm and neural crest using an *Ap2αcre* line (Macatee et al., 2003). As no defects were present in the neural crest-specific conditional knockouts, defects in the ectoderm + neural crest-specific gene deletion experiments can be ascribed to functions in the ectoderm. The developing facial nerve in *Ap2αcre*;*Spry1^f/–^*;*Spry2^f/–^* mutants showed a similar phenotype to *Spry1*^*−/−*^*;Spry2*^*−/−*^ embryos, where the geniculate ganglion was enlarged (n = 6/8, two tailed Fisher's exact test p = 0.0083) with abnormal nerve projections into the second arch (n = 3/8, two tailed Fisher's exact test p = 0.0364) (Table 1 and Fig. 5C). Curiously, most glossopharyngeal and all vagus nerves appeared normal in these mutants. *Ngn2* expression as a marker of epibranchial placodes at E9.5, confirmed that the geniculate placode was enlarged in these mutants (Figs. 5G,H). A slightly smaller petrosal placode was seen in *Ap2αcre*;*Spry1^f/–^*;*Spry2^f/–^* embryos (n = 2; Figs. 5G,H). These data suggest a cell autonomous role for *Spry1* and *Spry2* in the ectoderm during the development of the geniculate and to a lesser extent, the petrosal placode. To determine whether the enlarged geniculate placode was also associated with a larger second arch in *Ap2αcre*;*Spry1^f/–^*;*Spry2^f/–^* mutant embryos, the 2D lateral area of the second arch was measured. The second pharyngeal arch was not larger in these conditional mutants (n = 8, unpaired two tailed *t*-test p = 0.9579, Table 2), indicating that the enlarged geniculate is not due to a general enlargement of the 2nd arch. This observation indicates a cell autonomous role for Sprouty genes in the pharyngeal ectoderm during facial nerve development.

As signals from the pharyngeal endoderm have been implicated in placodal neurogenesis (Begbie et al., 1999; Nechiporuk et al., 2005), we also deleted *Spry1* and *Spry2* from this tissue using the *Sox17icre* line (Engert et al., 2009). Half of the facial nerves examined were defective in *Sox17icre;Spry1*^*f/f*^*;Spry2*^*f/f*^ embryos (n = 3/6, two tailed Fisher's exact test p = 0.0245). By contrast, the other branchial nerves appeared to develop normally in these conditional mutants (Fig. 5D). The geniculate placode was slightly larger and the petrosal slightly smaller in *Sox17icre;Spry1*^*f/f*^*;Spry2*^*f/f*^ mutant embryos (n = 4, Figs. 5I,J). These observations indicate that the loss of Sprouty gene function in the endoderm can affect the early development of the epibranchial placodes, albeit not as severely as when these genes are lost in all tissues. The 2D lateral area of the second arch in the *Sox17icre;Spry1*^*f/f*^*;Spry2*^*f/f*^ mutants (n = 12) was slightly increased compared to controls (n = 16) (unpaired two tailed *t*-test, p = 0.0352, Table 2), suggesting that the enlarged geniculate in these mutants might be secondary to arch defects.

Taken together, these observations suggest that defects in the pharyngeal endoderm caused by the deletion of Sprouty genes in the endoderm can have non cell-autonomous effects on the ectodermal placodes. Relatively small changes in *Ngn2* expression in the epibranchial placodes appear to be sufficient to cause some defects in the facial nerve, but insufficient to cause defects in nodose and vagus nerves.

### *Fgf8* haploinsufficiency partially rescues the *Spry1;Spry2* double knockout phenotype

**Fig. 6:**
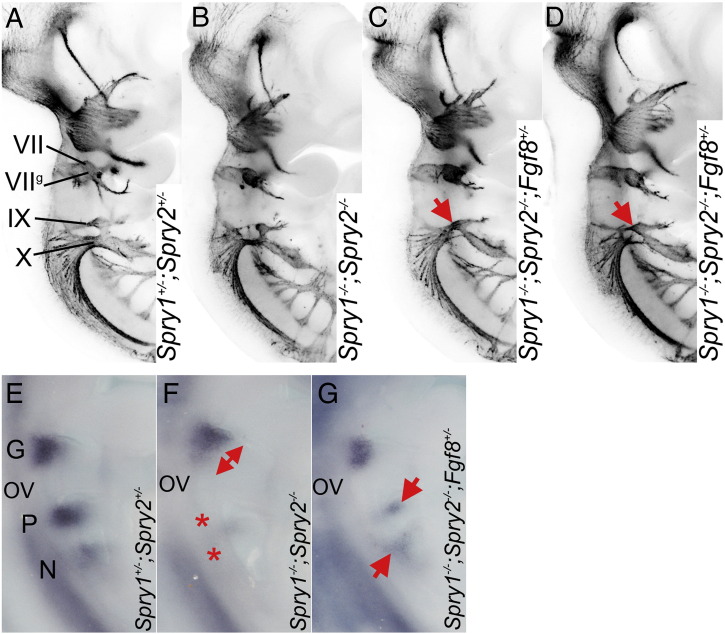
*Spry1−/−;Spry2−/−* epibranchial cranial nerve phenotype is partially rescued by *Fgf8* heterozygosity. (A–D) Anti-neurofilament immunohistochemistry showing the developing cranial nerves in *Spry1*^*+/−*^*;Spry2*^*+/−*^, *Spry1*^*−/−*^*;Spry2*^*−/−*^ and *Spry1*^*−/−*^*;Spry2*^*−/−*^;*Fgf8*^*+/−*^ embryos. Note the increased glossopharyngeal nerve fibres in *Spry1*^*−/−*^*;Spry2*^*−/−*^;*Fgf8*^*+/−*^ embryos (red arrows in C and D) compared to the *Spry1*^*−/−*^*;Spry2*^*−/−*^ embryo (B). (E–G) Epibranchial placodes in E9.5 embryos as revealed by a *Ngn2* antisense RNA probe. Note the enlarged geniculate (red arrow) and absence of petrosal and nodose placodes (red stars) in the *Spry1*^*−/−*^*;Spry2*^*−/−*^ embryo (F), and the rescue of these phenotypes in the *Spry1*^*−/−*^*;Spry2*^*−/−*^;*Fgf8*^*+/−*^ embryo, especially the presence of the petrosal and nodose placodes (red arrows in G).

Sprouty proteins regulate signalling via the MAPK pathway, which is activated downstream of a number of receptor tyrosine kinases, including FGF receptors and RET (Basson et al., 2005, 2006; Mason et al., 2006; Rozen et al., 2009; Shim et al., 2005). To determine to what extent the observed defects are due to hyperactive FGF signalling, we attempted to rescue the *Spry1*^*−/−*^*;Spry2*^*−/−*^ mutant phenotype by reducing the *Fgf8* gene dosage as previously described for *Spry2* mutant phenotypes (Shim et al., 2005). The epibranchial cranial nerves in *Spry1*^*−/−*^*;Spry2*^*−/−*^*;Fgf8*^*+/−*^ embryos (Figs. 6C,D) exhibited similar phenotypes to those observed in *Spry1*^*−/−*^*;Spry2*^*−/−*^ (Fig. 6B). However, whereas the incidence of phenotypes found in the developing facial and vagus cranial nerves were similar between *Spry1*^*−/−*^*;Spry2*^*−/−*^ and *Spry1*^*−/−*^*;Spry2*^*−/−*^*;Fgf8*^*+/−*^ embryos, the density of glossopharyngeal nerve fibres was rescued in some *Spry1*^*−/−*^*;Spry2*^*−/−*^*;Fgf8*^*+/−*^ embryos (Figs. 6C,D, Table 1). To explore the reason for this partial phenotypic rescue further, we compared the forming epibranchial placodes in *Spry1*^*−/−*^*;Spry2*^*−/−*^ and *Spry1*^*−/−*^*;Spry2*^*−/−*^*;Fgf8*^*+/−*^ embryos. Both the enlarged geniculate and absent petrosal and nodose phenotypes were partially rescued by a reduction in the *Fgf8* gene dosage at E9.5 (n = 4; Figs. 6E–G). This observation suggests that increased signalling downstream of FGF8 may be responsible for these early defects in placodal development.

## Discussion

In this manuscript we describe the analysis of branchial nerve phenotypes in embryos in which two Sprouty genes (*Spry1* and *Spry2*) were deleted. Our analysis indicates that the facial nerve is most sensitive to the loss of Sprouty genes with 97% of all facial nerves investigated being abnormal, compared to the glossopharyngeal (53%) and vagus (56%) nerves. We further show that the facial nerve phenotype could be observed upon the deletion of these genes from either the pharyngeal endoderm or ectoderm, whereas the other two nerves developed normally in these conditional mutants. Thus, our observations suggest that abnormal glossopharyngeal and vagus nerve development may require the deletion of Sprouty genes from several tissues at once. Finally, we present evidence that early epibranchial placode development is perturbed in Sprouty-deficient embryos due to hyperactive FGF8 signalling. Intriguingly, reducing the *Fgf8* gene dosage rescues these early phenotypes but is only sufficient to partially rescue the glossopharyngeal phenotype. This observation suggests that Sprouty proteins may also inhibit signalling downstream of other factors that regulate branchial nerve development. Multiple FGF ligands (FGF3, FGF8, FGF10 and FGF15) are expressed in the developing pharyngeal region and show considerable functional redundancy during otic development (Schimmang, 2007; Wright and Mansour, 2003). In addition, signals from other factors such as GDNF can also be regulated by Sprouty (Basson et al., 2005, 2006).

Our results indicate that the effects of deregulated RTK signalling on the early development of the geniculate placode differ from effects on the petrosal and nodose. These observations are consistent with findings in zebrafish, where it has been proposed that the mechanisms that induce formation of the facial and large vagal placodes differ from those that control glossopharyngeal and small vagal placode formation (Nechiporuk et al., 2005). We found that increased RTK signalling is associated with an enlarged geniculate placode, whereas the petrosal and nodose are smaller under these conditions. Our data are consistent with previous studies in the mouse embryo, in particular those that analysed the effects of reduced FGF signalling on pharyngeal development. Trokovic et al. have shown that several FGF genes, *Fgf3*, *Fgf8* and *Fgf15* are expressed in the developing second pharyngeal arch and that this arch is severely hypoplastic in *Fgfr1* hypomorphic mutants (Trokovic et al., 2005). Their data suggest that the second arch is most dependent upon FGF signalling for its development. Furthermore, the geniculate placode is also hypoplastic in these mutants, whereas the petrosal and nodose develop normally. Our data suggest that increased FGF signalling upon the deletion of Sprouty genes results in the opposite phenotype i.e. an enlarged second arch and geniculate placode. Thus, the facial nerve defects in Sprouty mutants could, at least in part, be explained by the unique sensitivity of the second arch to perturbations in FGF signalling. However, the geniculate, placode and facial nerve phenotypes were still observed in ectoderm/neural crest-specific conditional knockout embryos, despite the second arch being of normal size in these mutants. This observation suggests that these two phenotypes are not necessarily causally linked, and implies a cell-autonomous role for Sprouty genes in the pharyngeal ectoderm itself during geniculate placode specification, consistent with a model proposed by Trokovic et al. (Trokovic et al., 2005). Interestingly, our conditional gene deletion experiments suggest that the enlarged arch in the Sprouty mutants, might primarily be due to the loss of Sprouty genes in the endoderm.

Whereas the development of the posterior epibranchial placodes, the petrosal and nodose, is not affected when FGF signalling is reduced as in *Fgfr1* or *Fgf8* hypomorphs (Chi et al., 2003; Trokovic et al., 2005), we find that increased RTK signalling delays the formation of these placodes. A recent analysis of early otic specification in *Spry1−/−;Spry2−/−* embryos revealed that the *Pax2+;Pax8+;Dlx5+;Foxi2−* otic placode is expanded in these mutants, apparently at the expense of the *Foxi2+* non-otic ectoderm, which presumably includes precursors of the epibranchial placodes (Mahoney Rogers et al., 2011). Thus, the loss of Sprouty genes does not appear to affect the earlier steps of otic/epibranchial development, but promotes otic fate at the expense of non-otic ectoderm fate at later stages of development. Our genetic rescue experiments suggest that the defects in these two caudal epibranchial placodes are, at least in part, due to deregulated signalling downstream of *Fgf8*. As *Fgf8*, *Fgf3* and *Fgf10* are functionally redundant during specification of the pre-otic field and otic placode (Alvarez et al., 2003; Dominguez-Frutos et al., 2009; Ladher et al., 2005; Wright and Mansour, 2003; Zelarayan et al., 2007), one would predict that compound mutants in these genes would also exhibit defects in the branchial nerves. However, as these FGFs are required at an early stage of development for the specification of the otic-epibranchial progenitor domain, these mutants are likely to exhibit reduced specification of epibranchial placodes in addition to the already-described reduction in the size of the otic placode. Further work will be required to test this hypothesis.

Our analysis of neurogenesis in Sprouty-deficient embryos suggests that the differentiation of *Ngn2*-positive placodal cells into *NeuroD*-positive neuroblasts was inhibited by excessive RTK signalling. The reduction in *NeuroD* expression was consistent in all developing branchial ganglia. This defect was transient suggesting that neural differentiation was merely delayed for a short time. A transient delay, as opposed to a complete block in neural differentiation is consistent with the finding that the facial ganglion was bigger in Sprouty mutants, as the larger number of *Ngn2+* placodal precursors specified in these mutants, is expected to eventually contribute more neurons to this ganglion. FGF signals have been reported to inhibit neuronal differentiation in other developmental contexts, including the prospective chick spinal cord (Diez del Corral et al., 2002, 2003). Studies in the chick embryo have identified one potential regulator of epibranchial neurogenesis as BMP7 (Begbie et al., 1999). The expression of *Bmp7* was not significantly altered in *Fgfr1* hypomorphs (Trokovic et al., 2005), or *Spry1*^*−/−*^*;Spry2*^*−/−*^ mutants (this study, not shown), indicating that FGF signalling is unlikely to affect neurogenesis by controlling *Bmp7* gene expression. However, activated ERK/MAPK has been shown to inhibit BMP signalling at the level of Smad proteins in developmental contexts (Pera et al., 2003). Thus, increased RTK signalling in Sprouty mutants may inhibit the neurogenic activity of BMP7 through inhibiting Smad activity.

Chick and zebrafish experiments have shown that epithelial FGF signalling regulates neural crest cell fate decisions in the pharyngeal apparatus, promoting a ectomesenchymal fate over a neurogenic fate (Blentic et al., 2008). Our results suggest that this also applies to the mouse embryo. The neurogenic crest marker *Sox10* is downregulated and the ectomesenchymal neural crest marker, *Dlx2*, upregulated in regions of increased RTK signalling in Sprouty mutants.

In conclusion, previous studies have identified a number of different functions for FGF signalling in the pharyngeal region. Data presented in this manuscript demonstrate that the RTK antagonists, Sprouty1 and Sprouty2 are essential for the normal development of the branchial nerves. The loss of *Spry1* and *Spry2* results in a number of defects, including an enlargement of the second pharyngeal arch, defects in epibranchial placode formation and alterations in neural crest fate. Our attempts to dissect the tissue-specific requirements of Sprouty gene function in these processes showed that the second arch expansion is at least partially due to the loss of Sprouty genes in the endoderm. Finally, the expansion of the geniculate placode and facial nerve dysmorphology are due to the loss of Sprouty genes from the pharyngeal endoderm and ectoderm. Thus, a rather complicated picture emerges that suggests that Sprouty gene function is required in several cell types and that the branchial nerve phenotypes observed in Sprouty-deficient embryos cannot be explained by Sprouty loss within a single tissue or cell type.

The following are the supplementary materials related to this article.

## Figures and Tables

**Table 1:** Branchial nerve phenotypes in E10.5 embryos with indicated genotypes.

	VII	IX	X	Total
Wildtype	0	0	0	10
*Spry1*^*+/−*^*;Spry2*^*+/−*^	6	2	2	34
*Spry1*^*−/−*^	0	0	1	10
*Spry2*^*−/−*^	0	0	0	8
*Spry1*^*−/−*^*;Spry2*^*−/−*^	35⁎⁎	19⁎⁎	20⁎⁎	36
*Fgf8*^*+/−*^	0	0	0	8
*Spry1*^*−/−*^*;Spry2*^*−/−*^*;Fgf8*^*+/−*^	4	0	4	4
*Ap2αcre;Spry1*^*f/+*^*;Spry2*^*f/+*^	2	1	0	14
*Ap2αcre;Spry1*^*f/−*^*;Spry2*^*f/−*^	6⁎⁎	1	0	8
*Wnt1cre;Spry1*^*f/+*^*;Spry2*^*f/+*^	0	0	0	8
*Wnt1cre;Spry1*^*f/−*^*;Spry2*^*f/−*^	0	0	0	8
*Sox17icre;Spry1*^*f/+*^*;Spry2*^*f/+*^	0	0	0	12
*Sox17icre;Spry1*^*f/f*^*;Spry2*^*f/f*^	3⁎	0	0	6

The number of defective nerves is recorded for each genotype, along with the total number of nerves analysed. Statistical analysis was performed using Prism 5 (Graphpad software Inc). Two-tailed Fisher's exact test was used to calculate the probability of a significant difference. For statistical analyses, Spry1^−/−^, Spry2^−/−^ and Spry1^+/−^;Spry2^+/−^ embryos were compared with wildtype, *Spry1*^*−/−*^*;Spry2*^*−/−*^ with Spry1^+/−^;Spry2^+/−^ and *Xcre;Spry1*^*f/f*^*;Spry2*^*f/f*^ or *Xcre;Spry1*^*f/−*^*;Spry2*^*f/−*^ with *Xcre;Spry1*^*f/+*^*;Spry2*^*f/+*^ embryos.

⁎ p < 0.05.

⁎⁎ p < 0.01.

**Table 2:** 2D lateral pharyngeal arch area standardised for the height of the embryo at E9.5.

Genotype	Number	First pharyngeal arch (StD)	Second pharyngeal arch (StD)
*Spry1*^*+/−*^*;Spry2*^*+/−*^	36	11114 (1198)	7056 (894.1)
*Spry1*^*−/−*^*;Spry2*^*−/−*^	36	11532 (1486)	9040** (1266)
*Ap2αcre;Spry1*^*f/+*^*;Spry2*^*f/+*^	4	11275 (1110)	6420 (668.5)
*Ap2αcre;Spry1*^*f/−*^*;Spry2*^*f/−*^	4	9652 (1556)	6441 (333.4)
*Wnt1cre;Spry1*^*f/+*^*;Spry2*^*f/+*^	8	10922 (796.7)	7091 (682.6)
*Wnt1cre;Spry1*^*f/−*^*;Spry2*^*f/−*^	8	11284 (775.8)	7410 (637.4)
*Sox17cre;Spry1*^*f/+*^*;Spry2*^*f/+*^	16	10652 (847.9)	6836 (772.1)
*Sox17cre;Spry1*^*f/−*^*;Spry2*^*f/−*^	12	10912 (1200)	7628* (1036)

The 2D lateral pharyngeal arch area was measured using Adobe Photoshop (Adobe Systems Incorporated) and standardised to the length of the embryo (Suppl. Fig. 2). Statistical significant differences were calculated using Prism 5 (Graphpad software Inc). With the exception of a single (underlined) group, all passed the D'Agostino and Pearson omnibus normality test and were analysed using the unpaired two-tailed *t*-test, *p < 0.05 and **p < 0.01. Two-tailed Mann–Whitney test was used for the measurement set that failed the normality test. *Spry1*^*−/−*^*;Spry2*^*−/−*^ embryos were compared with Spry1^+/−^;Spry2^+/−^ and *Xcre;Spry1*^*f/f*^*;Spry2*^*f/f*^ or *Xcre;Spry1*^*f/−*^*;Spry2*^*f/−*^ with *Xcre;Spry1*^*f/+*^*;Spry2*^*f/+*^embryos for the purpose of statistical analysis.
